# Supplementary material for: Short communication: Lifetime musical activity and resting-state functional connectivity in cognitive networks
Source: PLoS One. 2024 May 2;19(5):e0299939. doi: 10.1371/journal.pone.0299939 (PMC11065262; doi:10.1371/journal.pone.0299939)
Supplement: S1 File — (DOCX) [file pone.0299939.s001.docx]

**Supplement:**

**Lifetime musical activity and resting-state functional connectivity in cognitive networks**

## Supplementary methods

### Participant selection procedure

In the present study, we selected cognitively unimpaired participants from the DELCODE baseline cohort, i.e., healthy controls (HC), participants with subjective cognitive decline (SCD), and Alzheimer’s disease (AD) family history (FH), that had MRI data available (see **S1 Fig.**). In the next steps, participants were filtered according to the availability of lifetime musical activity data (explained further below), relevant covariates, and good-quality functional MRI data (total: *n =* 394), leaving two groups of participants (participants with musical activity: *n =* 65, controls: *n =* 329). These two groups were matched using the one-to-one matching procedure based on the R package *MatchIt* (version 4.1.0) [1] and the nearest-neighbor method. We considered socioeconomic status (SES), crystallized intelligence, self-reported physical activity, sex, age, years of education, and diagnostic group as matching variables. The final sample included 130 participants (participants with musical activity: *n =* 65, matched controls: *n =* 65). Note, an overlapping sample was used in our previous study [2].

**Insert about here: *See additional image file (Fig_S1)***

***
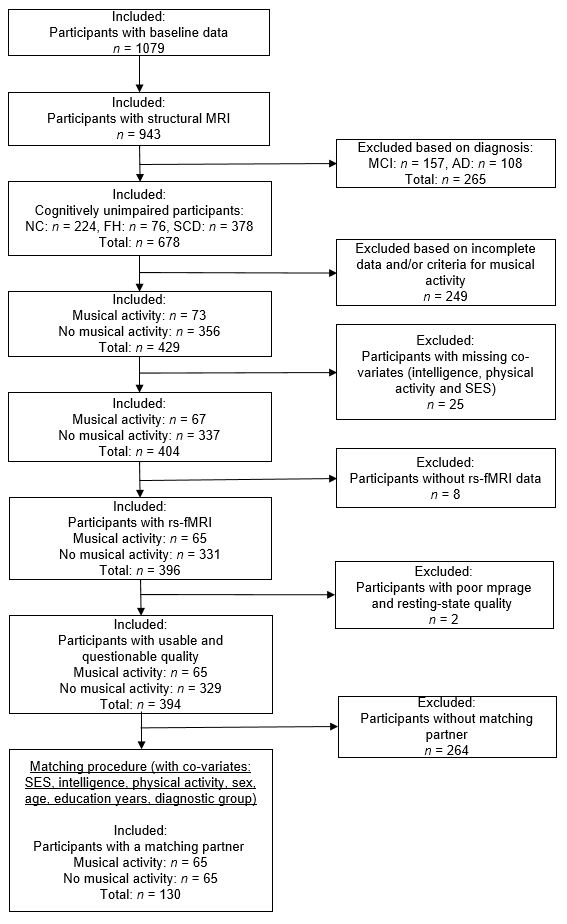
***

**S1 Fig. Selection flow chart.** The baseline DELCODE dataset included 1079 participants, and only participants with structural MRI data were included in the further selection process (n = 943). This study examined cognitively unimpaired participants; therefore, participants with mild cognitive impairment (MCI) and Alzheimer’s disease (AD) were excluded. In the next steps, only participants with complete data on lifetime musical activity assessment, matching variables, and covariates were included (total: n = 404). Finally, participants with available rs-fMRI and good-quality image data were considered for the matching process (total: n = 394). The final sample after the matching process consisted of n = 130 participants (participants with lifetime musical activity: n = 65, controls: n = 65). FH: family history of Alzheimer’s disease, MRI: magnetic resonance imaging, NC: normal cognition (healthy controls), rs-fMRI: resting-state functional magnetic resonance imaging, SCD: subjective cognitive decline, SES: socioeconomic status.

### Assessment of musical activity

Lifetime musical activity was assessed using the Lifetime of Experiences Questionnaire (LEQ) [3] in a version adapted to the German population (LEQ-D) [4]. Participants answered questions about the frequency of playing a musical instrument (“How often did you play a musical instrument?”) over their lifetime (for the life stages 13-30 years, 30-65 years, 65 years and older) on a 6-point Likert-scale (0: never, 1: less than once a month, 2: once a month, 3: twice a month, 4: once a week, 5: daily).

We evaluated musical activity as a binary group variable based on a coding scheme developed in our previous study [2]. The group with lifetime musical activity (*n =* 65) included participants, who reported having played a musical instrument in each life stage (i.e., no life stage included ‘never’) and with high frequency (≥ 2 times per month) in at least one life stage. The control group (*n =* 65) included participants, who reported to never have played a musical instrument in any life stage (i.e., all life stages included ‘never’). To ensure that there were no false classifications, participants with missing responses on the musical activity item in any life stage (taking into account their respective age) were excluded.

**Insert about here: *See additional image file (Fig_S2)***

***
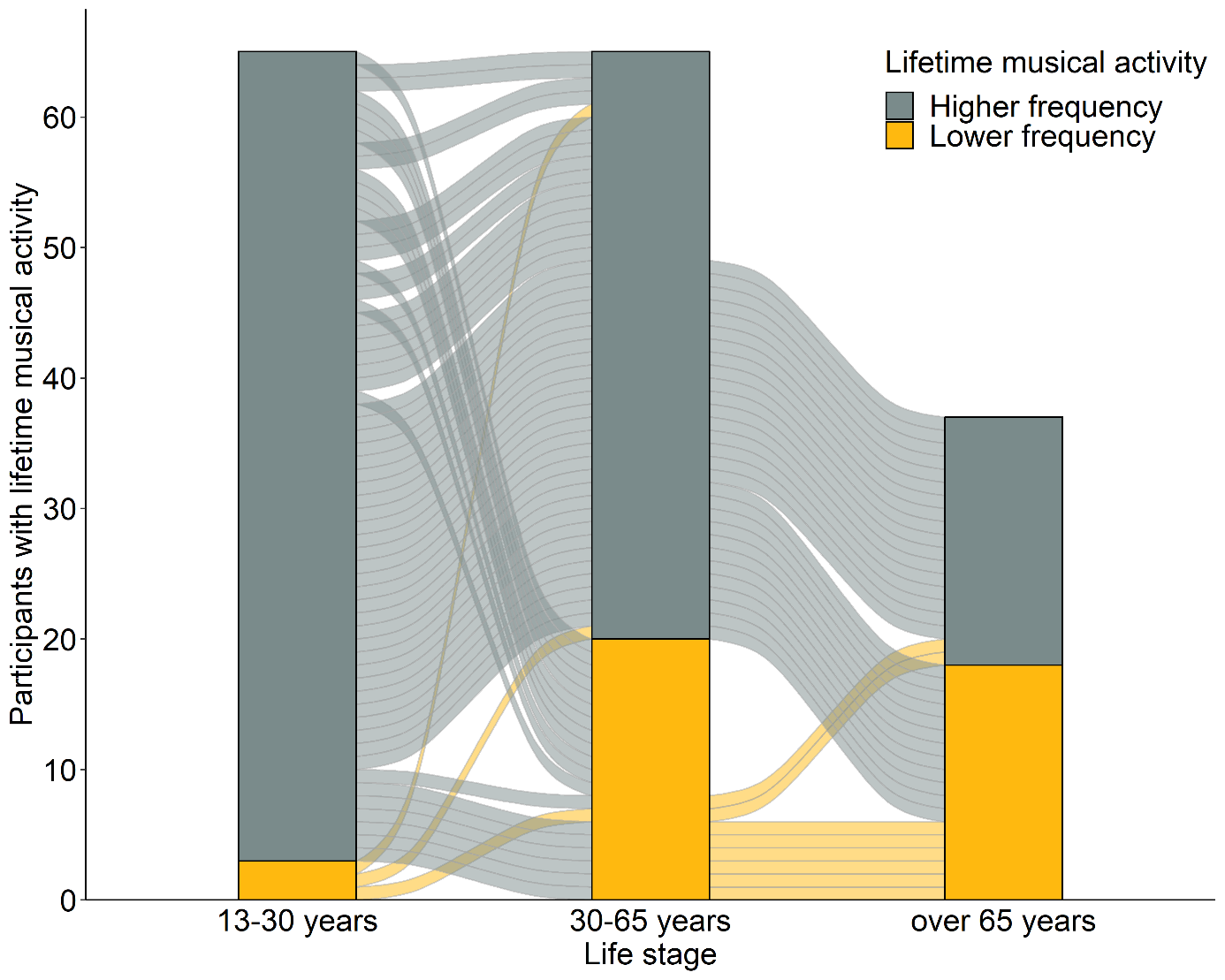
***

**S2 Fig. Descriptive characterization of the included participants (n = 65) with lifetime musical activity across the three life stages.** Information is based on participants’ responses about the frequency of musical activity in the Lifetime of Experiences Questionnaire (LEQ). The assessment of lifetime musical activity was - dependent on the participants’ age - assessed across two life stages (13-30 years and 30-65 years) or across three life stages (13-30 years, 30-65 years, 65 years and older).

### Acquisition of neuroimaging data

The magnetic resonance imaging (MRI) data were acquired with nine 3.0 Tesla Siemens scanner (one Prisma system, one Skyra system, three TIM Trio systems, four Verio systems) at nine DZNE sites [5]. For this study, we used structural T1-weighted (isotropic voxel size 1 mm, matrix size 256×256, 192 slices, echo time 4.37 ms, repetition time 2500 ms, flip angle 7°, parallel imaging acceleration factor 2) acquired in a 5 min magnetization-prepared rapid gradient echo (MPRAGE) and resting-state functional MRI (rs-fMRI) data (isotropic voxel size 3.5 mm, matrix size 64×64, 47 slices, 180 volumes, echo time 30 ms, repetition time 2580 ms, flip angle 80°, parallel imaging acceleration factor 2) acquired in 7 min and 54 s [5]. All images were subsequently subjected to quality assurance by the DZNE Magdeburg.

### Assessment of physical activity

Lifetime physical activity was assessed based on the participants’ responses on questions about the frequency of physical activity during life (at life stages 13-30 years, 30-65 years, ≥ 65 years) in the Lifetime of Experiences Questionnaire (LEQ) [3]. Because not all participants were 65 years of age and older, we included only the responses on frequency of physical activity for the 13-30 years and 30-65 years’ life stages in the mean calculation. Participants without data for these two life stages were excluded during the sample selection process. Current physical activity was measured using the Physical Activity Scale for the Elderly (PASE) [6]. The total PASE score was calculated based on the frequency, amount, and duration of leisure-time, work, and household activities over the past seven days. A higher total score indicates greater levels of current physical activity.

### Local and global functional connectivity

Local and global resting-state functional connectivity (RSFC) was determined using the Montreal Neurological Institute (MNI) coordinates of regions-of-interests (ROIs) implemented in CONN (**S1 Table**).

| ***S1 Table.*** *Network-specific ROIs with their MNI coordinates for the calculation of the global RSFC (for graphical representation see S3 Fig.).* | | |
| --- | --- | --- |
| Resting-state Network (RSN) | ROIs | MNI center-of-gravity coordinates  (x, y, z) |
|  |  |  |
| Default Mode Network (DMN) | Medial prefrontal cortex (MPFC) | +1, +55, -3 |
|  | Posterior cingulate cortex (PCC) | +1, -61, +38 |
|  | Left lateral parietal (LP) | -39, -77, +33 |
|  | Right lateral parietal (LP) | +47, -67, +29 |
| Fronto-Parietal Network (FPN) | Left lateral prefrontal cortex (LPFC) | -43, +33, +28 |
|  | Right lateral prefrontal cortex (LPFC) | +41, +38, +30 |
|  | Left posterior parietal cortex (PPC) | -46, -58, +49 |
|  | Right posterior parietal cortex (PPC) | +52, -52, +45 |
| Salience Network (SAL) | Anterior cingulate cortex (ACC) | 0, +22, +35 |
|  | Left anterior insula (AInsula) | -44, +13, +1 |
|  | Right anterior insula (AInsula) | +47, +14, 0 |
| MNI: Montreal Neurological Institute; ROIs: regions-of-interest; RSFC: resting-state functional connectivity. | | |

## Supplementary results

**Insert about here: *See additional image file (Fig_S3)***

***
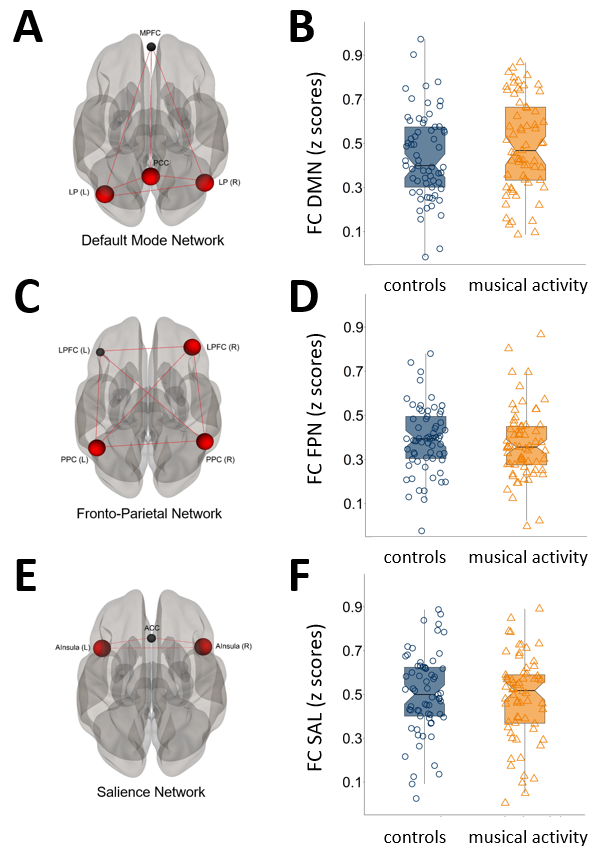
***

***S3 Fig. Result of the global (within-network) connectivity analysis.*** *The ROI-to-ROI connectivity analysis within all three RSN showed no significant group differences. The brain maps* ***(left side)*** *show the DMN* ***(A.)****, the FPN* ***(C.)*** *and the SAL* ***(E.)*** *networks with all investigated ROIs. Note, ROIs displayed in dark grey specify the network seeds used in our local (seed-to-voxel) connectivity analysis. The corresponding graphs* ***(right side)*** *show the non-significant difference between older participants with lifetime musical activity and matched controls in their global resting-state connectivity (z scores) within the DMN* ***(B.)****, the FPN* ***(D.)*** *and the SAL* ***(F.)****. Box plots display the median with 95% confidence intervals, interquartile range with lower (25th) and upper percentiles (75th), and individual data points within each group.* ***Key:*** *ACC: Anterior Cingulate Cortex, AInsula (L): Anterior Insula left, AInsula (R): Anterior Insula right, DMN: Default Mode Network, FC: Functional connectivity, FPN: Fronto-Parietal Network, LP (L): Lateral parietal left, LP (R): Lateral parietal right, LPFC (L): Lateral Prefrontal Cortex left, LPFC (R): Lateral Prefrontal Cortex right, MPFC: Medial Prefrontal Cortex, ROI: Regions-of-interest, PCC: Posterior Cingulate Cortex, PPC (L): Posterior Parietal Cortex left, PPC (R): Posterior Parietal Cortex right, RSN: Resting-State Networks, SAL: Salience Network.*

## References

1. Ho DE, Imai K, King G, Stuart EA. MatchIt: Nonparametric preprocessingfor parametric causal inference. Journal of Statistical Software. 2011;42(i08).

2. Böttcher A, Zarucha A, Köbe T, Gaubert M, Höppner A, Altenstein S, et al. Musical Activity During Life Is Associated With Multi-Domain Cognitive and Brain Benefits in Older Adults. Front Psychol. 2022;13:945709.

3. Valenzuela MJ, Sachdev P. Assessment of complex mental activity across the lifespan: development of the Lifetime of Experiences Questionnaire (LEQ). Psychol Med. 2007;37(7):1015-25.

4. Roeske S, Wolfsgruber S, Kleineidam L, Zulka L, Buerger K, Ewers M, et al. P3-591: A German version of the lifetime of experiences questionnaire (LEQ) to measure cognitive reserve: Validation results from the DELCODE study. Alzheimer's & Dementia. 2018;14(7S_Part_25):P1352-P3.

5. Jessen F, Spottke A, Boecker H, Brosseron F, Buerger K, Catak C, et al. Design and first baseline data of the DZNE multicenter observational study on predementia Alzheimer's disease (DELCODE). Alzheimers Res Ther. 2018;10(1):15.

6. Washburn RA, McAuley E, Katula J, Mihalko SL, Boileau RA. The Physical Activity Scale for the Elderly (PASE): Development and evaluation. Journal of Clinical Epidemiology. 1993;46:153-62.
