# Supplementary material for: Short communication: Lifetime musical activity and resting-state functional connectivity in cognitive networks
Source: PLoS One. 2024 May 2;19(5):e0299939. doi: 10.1371/journal.pone.0299939 (PMC11065262; doi:10.1371/journal.pone.0299939)
Supplement: Attachment — Submitted filename: PONE-D-23-20713_reviewer_REVIEWED.pdf [file pone.0299939.s002.pdf]

RE: PONE-D-23-20713

The current manuscript describes an interesting and relevant cross-sectional study investigating the relationship between lifetime musical activity and resting-state functional connectivity (RSFC) in higher-order cognitive networks in the brain (i.e., the default mode network, frontoparietal, and salience networks).

Specifically, Dr. Liebscher and colleagues assessed n=130 cognitive unimpaired older adult individuals (age=>60 years) from the longitudinal DZNE-DELCODE cohort study on risk for dementia and cognitive decline. Individuals, who reported to have participated in musical instrument playing during early, middle, and late life stages (n=65) were compared to matched control subjects, who reported never to have played a musical instrument (n=65), in seed-to-voxel and within-networks RSFC patterns. Individuals with lifetime musical activity displayed higher local RSFC between medial prefrontal cortex, temporal, and frontal regions (right temporal pole and right precentral gyrus) versus control subjects.

The authors conclude that playing a musical instrument during life is related to a higher RSFC of medial prefrontal cortex with brain regions involved in higher-order cognitive and motor processes.

The manuscript is well-written and relevant. I only have the following minor points:

**Abstract:**

- Since it is indeed a strength and relevant for interpretation of the observed findings that the two participant groups were matched on several demographic factors, please write these out already in the abstract (i.e., highlight that groups were matched on: age, sex, educational years, crystallized intelligence etc. (cf., Table 1))

**Methods, 2.1 Participants:**

- Please provide details on how you specifically operationalized “cognitively unimpaired”? It is unclear how this was assessed with standard neuropsychological tests. Providing information on the criteria for fulfilling this categorization (e.g., performing within certain cut-off values) would be beneficial.

**Methods, 2.2 Measurements, 2.2.1 Self-reported assessment of musical activity:**

- Although detailed information on the specific categorization process and the one-to-one matching procedure are provided in another REF and in the Supplementary Material, the manuscript would benefit from including these descriptions in the main text. Currently, it is unclear how often participants were to have played a musical instrument during life stages to be categorized as ‘having a lifetime history of musical activity’ (i.e., their musical activity ‘frequency’) from the

only brief description provided in the current version of chapter 2.2.1. Please include these details from the reference and the supplementary materials here to enhance clarity. Indeed, this would equip the reader with enhanced ability to derive more nuanced conclusions of the identified association.

**Methods, 2.2.4 Additional measures:**

- How did you specifically operationalize socioeconomic status? Currently, it only says that SES was assessed using the International Socioeconomic Index of Occupational Information. Please specify what the specific items measure.

**Discussion:**

- Although the authors have ensured that there were no differences between musical activity participants and matched controls on several demographic variables (cf., Table 1), a more extensive examination of other unaccounted variables in the RSFC association analyses would be beneficial. Please provide a more comprehensive discussion of the potential factors that could also be linked to a history of musical activity and might have influenced the observed differences in local and global functional connectivity patterns (such as potential differences in functional capacity including autonomy, social activity/interpersonal relationships, and occupational functioning that may not be captured by the SES measure applied).

- Did the authors consider running sensitivity analyses with respect to individuals, who have reported only engaging in solo instrument performance, as compared to participation in musical bands as part of their lifetime musical activities? It would be of interest to explore this aspect and/or discuss the plausible associations potentially emanating from differences in musical instrument engagement on the identified neural benefits associated with participating in musical activities throughout life in older age.

- Additionally, it would be interesting to assess and/or discuss whether differences would emerge when accounting for the specific musical instruments played by the individuals (e.g., potential disparities between individuals engaged solely in vocal performance/singing, individuals exclusively playing instrument like the piano or violin, which additionally require intricate fine motor skills involving both the left and right hands). This would be particularly interesting with regards to the identified nodes of precentral gyrus and superior frontal gyrus found to be involved in motor processing and motor control tasks (cf., lines 362-365).

- Please also highlight that the samples sizes were modest and the findings need replication before firm conclusions can be drawn (before specifically highlighting the need for future longitudinal studies)

**Figure 1**

- In Figure 1C (FC cluster 1) and 1E (FC cluster 2), please provide value units on the Y-axis (currently, only values are reported)
