# Supplementary material for: Short communication: Lifetime musical activity and resting-state functional connectivity in cognitive networks
Source: PLoS One. 2024 May 2;19(5):e0299939. doi: 10.1371/journal.pone.0299939 (PMC11065262; doi:10.1371/journal.pone.0299939)
Supplement: Attachment — Submitted filename: review comments.docx [file pone.0299939.s003.docx]

The authors investigated the impact of playing musical instruments on the functional connectivity of cognitive neural networks. Utilizing the DELCODE Study database and employing a one-to-one matching procedure based on described characteristics, the authors compared FPN, DMN, SAL differences, revealing that "Higher RSFC has been related to protective lifestyle factors and preserved cognitive abilities in the context of brain pathology, supporting a contribution of these neural correlates to cognitive reserve". As suggested by the authors, the higher RSFC discovered in this study between higher-order cognitive and motor processes may represent neuroprotective correlates underlying musical activity in older adults (OA). However, the study design exhibits certain shortcomings. If possible, it is strongly recommended that the authors supplement essential data.

1. The issue of sample representativeness is raised. The text only mentions a one-to-one matching procedure, which introduces a considerable arbitrary selection component. It is strongly advised that the authors employ a 1:1 randomization approach to select samples, thus better eliminating differences in confounding factors (including those mentioned in the text, but not limited to only those) between the two groups.

2. It is strongly recommended that the authors supplement data on cognitive function, executive function, emotional changes, and relevant behavioral differences between the two groups in addition to the differences in resting-state brain functional connectivity. Calculating the correlation between brain functional connectivity differences and behavioral differences and conducting mediation analysis models or pathway analysis is preferable to establish persuasive evidence. It is crucial to demonstrate the specific aspects of cognitive function differences caused by the impact of playing musical instruments on the functional connectivity of cognitive neural networks. Therefore, the manuscript requires substantial data supplementation for a major revision.

3. The study lacks strict control over confounding factors. In addition to the factors mentioned in the text such as Age, Education, Crystallized Intelligence, Sex, Diagnostic group, Lifetime Physical Activity, Current Physical Activity, and LEQ, there may be other potential factors such as underlying diseases, nutritional status, emotional conditions, interpersonal relationships, etc. Adhering to the principle of random sampling is essential, and the sample size should ideally reach several thousand individuals.
