# Supplementary material for: Short communication: Lifetime musical activity and resting-state functional connectivity in cognitive networks
Source: PLoS One. 2024 May 2;19(5):e0299939. doi: 10.1371/journal.pone.0299939 (PMC11065262; doi:10.1371/journal.pone.0299939)
Supplement: Attachment — Submitted filename: Response_to_reviewers.docx [file pone.0299939.s004.docx]

# Response to Reviewers

## General comment to the reviewers and the academic editor

## We thank the academic editor and reviewers for taking the time to read our manuscript and providing us with comments that have helped improve our work. With the assistance of the reviewers' feedback, we have revised our manuscript and incorporated current literature. The modifications are highlighted in yellow within the "Revised Manuscript with Changes Highlighted" document and are also referenced in our responses here.

## Academic editor comments

#### General comment

**“First, let me apologize on behalf of Plos ONE for the time needed to review this manuscript. It was very difficult to secure reviewers, also because a few reviewers with specific experience in the field were in conflict of interest.**

**Reviewers 2 and 3 made a number of methodological comments that authors must consider, but not necessarily address by changing the processing strategy or study design. In particolar, I think that the "matched pair" experimental design is acceptable in this context; probably authors, rather than changing study design, can discuss the limitations of the approach in the relavant section of the manuscript.**

**Thank you for submitting your manuscript to PLOS ONE. After careful consideration, we feel that it has merit but does not fully meet PLOS ONE’s publication criteria as it currently stands. Therefore, we invite you to submit a revised version of the manuscript that addresses the points raised during the review process.”**

**Response:** We thank the academic editor for their time, patience and hard work in finding reviewers for our manuscript. We have addressed all of the reviewers' comments in this document and have revised our manuscript accordingly. We particularly appreciate your guidance regarding the critique of our matched-pair study design. We will also address all the points you raised below.

#### All comments

##### Comment #1

**“1. Please ensure that your manuscript meets PLOS ONE's style requirements, including those for file naming. The PLOS ONE style templates can be found at**

**https://journals.plos.org/plosone/s/file?id=wjVg/PLOSOne_formatting_sample_main_body.pdf and**

**https://journals.plos.org/plosone/s/file?id=ba62/PLOSOne_formatting_sample_title_authors_affiliations.pdf“**

Response: We thank you for bringing this to our attention and referring us to this helpful documentation. Based on these documents, we have changed the format and style of the headings, changed the naming of the figures in the text, modified the affiliation of the authors, and changed the names of the files. We have also ensured that we use one of the citation styles required by PLOS One (Vancouver).

##### Comment #2

**“2. Did you know that depositing data in a repository is associated with up to a 25% citation advantage (https://doi.org/10.1371/journal.pone.0230416)? If you’ve not already done so, consider depositing your raw data in a repository to ensure your work is read, appreciated and cited by the largest possible audience. You’ll also earn an Accessible Data icon on your published paper if you deposit your data in any participating repository (**[**https://plos.org/open-science/open-data/#accessible-data)**](https://plos.org/open-science/open-data/#accessible-data))**.”**

##### Response: We thank you for sharing this interesting fact, but our data is particularly sensitive and personal data, which is why we cannot make it publicly accessible. However, it can be made accessible upon reasonable request. We have also uploaded our R scripts that were used for the data analyses to the open science framework.

**Quote from online submission form and our manuscript on page 28:** “Due to the sensitive and personal nature of the data, we are unable to publicly disclose the data. The anonymized data used for this study will be made available by request from any qualified investigator through the DZNE-DELCODE Steering Board for purposes of replicating procedures and results. Requests to access the minimal dataset should be directed to the German Center for Neurodegenerative Diseases (DZNE), Bonn. For contact information please refer to: <https://www.dzne.de/en/research/studies/clinical-studies/delcode/> (Study Coordination and Project Management). We used existing data analysis packages for the neuroimaging and statistical analysis of this study. All relevant R scripts used for data analysis are freely available via the open science framework (OSF, https://osf.io/g73a9/).”

##### Comment #3

**“3. We note that the grant information you provided in the ‘Funding Information’ and ‘Financial Disclosure’ sections do not match. When you resubmit, please ensure that you provide the correct grant numbers for the awards you received for your study in the ‘Funding Information’ section.”**

##### Response: We appreciate you bringing this matter to our attention. Indeed, it seems there has been a misunderstanding, and we apologize for any confusion. The funding information referenced in your comment #4 does pertain to our competing interest. We would therefore be very grateful if the funding information in the online submission form could be replaced by the following statement:

**Correct funding statement:** “The overall DELCODE study was funded by the German Center for Neurodegenerative Diseases (Deutsches Zentrum für Neurodegenerative Erkrankungen [DZNE]), reference number: BN012. For the present study, the authors received no specific funding. The funders had no role in study design, data collection and analysis, decision to publish, or preparation of the manuscript.”

##### Comment #4

**„4. Thank you for providing the following Funding Statement:**

**“O. Peters received fees for consultation from Abbvie, Biogen, Eisai, Griffols, MSD Roche, and Schwabe. J. Priller received fees for consultation, lectures, and patents from Neurimmune, Axon, Desitin, and Epomedics. J. Wiltfang is an advisory board member of Abbott, Biogen, Boehringer Ingelheim, Immunogenetics, Lilly, MSD Sharp & Dohme, and Roche Pharma and received honoraria for lectures from Actelion, Amgen, Beeijing Yibai Science and Technology Ltd., Janssen Cilag, Med Update GmbH, Pfizer, Roche Pharma and holds the following patents: PCT/EP 2011 001724 and PCT/EP 2015 052945. J. Wiltfang is supported by an Ilidio Pinho professorship, iBiMED (UIDB/04501/2020) at the University of Aveiro, Portugal. E. Düzel received fees for consultation from Roche, Biogen, RoxHealth and holds shares in neotiv. F. Jessen received fees for consultation from Eli Lilly, Novartis, Roche, BioGene, MSD, Piramal, Janssen, and Lundbeck. The remaining authors report no conflicts of interest.”**

**We note that one or more of the authors is affiliated with the funding organization, indicating the funder may have had some role in the design, data collection, analysis or preparation of your manuscript for publication; in other words, the funder played an indirect role through the participation of the co-authors. If the funding organization did not play a role in the study design, data collection and analysis, decision to publish, or preparation of the manuscript and only provided financial support in the form of authors' salaries and/or research materials, please review your statements relating to the author contributions, and ensure you have specifically and accurately indicated the role(s) that these authors had in your study in the Author Contributions section of the online submission form. Please make any necessary amendments directly within this section of the online submission form. Please also update your Funding Statement to include the following statement: “The funder provided support in the form of salaries for authors [insert relevant initials], but did not have any additional role in the study design, data collection and analysis, decision to publish, or preparation of the manuscript. The specific roles of these authors are articulated in the ‘author contributions’ section.” If the funding organization did have an additional role, please state and explain that role within your Funding Statement.**

**Please also provide an updated Competing Interests Statement declaring this commercial affiliation along with any other relevant declarations relating to employment, consultancy, patents, products in development, or marketed products, etc.**

**Within your Competing Interests Statement, please confirm that this commercial affiliation does not alter your adherence to all PLOS ONE policies on sharing data and materials by including the following statement: "This does not alter our adherence to PLOS ONE policies on sharing data and materials.” (as detailed online in our guide for authors http://journals.plos.org/plosone/s/competing-interests). If this**

**adherence statement is not accurate and there are restrictions on sharing of data and/or materials, please state these. Please note that we cannot proceed with consideration of your article until this information has been declared.”**

##### Response: We are deeply appreciative of your comment. As previously explained in comment #3, the funding statement you referenced pertains to our competing interest. We apologize for any misunderstanding. The correct representation of our funding statement is as follows and we would be grateful if this could be corrected in the online submission form. In response to your comment, we have added a sentence regarding the role of the funder in this study.

**Correct funding statement:** “The overall DELCODE study was funded by the German Center for Neurodegenerative Diseases (Deutsches Zentrum für Neurodegenerative Erkrankungen [DZNE]), reference number: BN012. For the present study, the authors received no specific funding. The funders had no role in study design, data collection and analysis, decision to publish, or preparation of the manuscript.”

We have also updated our competing interest as follows and would be grateful if this could also be updated on the online submission form:

**Correct competing interest statement:** “O. Peters received fees for consultation from Abbvie, Biogen, Eisai, Griffols, MSD Roche, and Schwabe. J. Priller received fees for consultation, lectures, and patents from Neurimmune, Axon, Desitin, and Epomedics. J. Wiltfang is an advisory board member of Abbott, Biogen, Boehringer Ingelheim, Immunogenetics, Lilly, MSD Sharp & Dohme, and Roche Pharma and received honoraria for lectures from Actelion, Amgen, Beeijing Yibai Science and Technology Ltd., Janssen Cilag, Med Update GmbH, Pfizer, Roche Pharma and holds the following patents: PCT/EP 2011 001724 and PCT/EP 2015 052945. J. Wiltfang is supported by an Ilidio Pinho professorship, iBiMED (UIDB/04501/2020) at the University of Aveiro, Portugal. E. Düzel received fees for consultation from Roche, Biogen, RoxHealth and holds shares in neotiv. F. Jessen received fees for consultation from Eli Lilly, Novartis, Roche, BioGene, MSD, Piramal, Janssen, and Lundbeck. The remaining authors report no conflicts of interest. This does not alter our adherence to PLOS ONE policies on sharing data and materials. “

##### Comment #5

**“5. PLOS requires an ORCID iD for the corresponding author in Editorial Manager on papers submitted after December 6th, 2016. Please ensure that you have an ORCID iD and that it is validated in Editorial Manager. To do this, go to ‘Update my Information’ (in the upper left-hand corner of the main menu), and click on the Fetch/Validate link next to the ORCID field. This will take you to the ORCID site and allow you to create a new iD or authenticate a pre-existing iD in Editorial Manager. Please see the following video for instructions on linking an ORCID iD to your Editorial Manager account: https://www.youtube.com/watch?v=_xcclfuvtxQ.”**

##### Response: We thank you for this information. We have added the ORCID iD to the PLOS ONE profile of the corresponding author.

##### Comment #6

**“6. Your ethics statement should only appear in the Methods section of your manuscript. If your ethics statement is written in any section besides the Methods, please delete it from any other section.”**

**Response:** We thank you for this comment and have included the ethics statement with the relevant information in our methods section and removed it from all other sections.

**Quote from the manuscript, p. 6:** “This study was based on the baseline dataset of the DZNE–DELCODE cohort, an ongoing German multicenter longitudinal study [32] designed and carried out in accordance with the ethical principles of the Declaration of Helsinki. The protocol was approved by local ethical committees at each study site and all participants provided written informed consent.”

**Comment #7**

**“7. Please include captions for your Supporting Information files at the end of your manuscript, and update any in-text citations to match accordingly. Please see our Supporting Information guidelines for more information: http://journals.plos.org/plosone/s/supporting-information.”**

**Response:** We thank you for this information. We have added the Supporting Information section to the manuscript as follows.

**Quote from the manuscript, p. 21: “S1 File. Supplement: Lifetime musical activity and resting-state functional connectivity in cognitive networks.** Contains supplementary methods and results as well as the following:

**S1 Fig. Selection flow chart of our sample.** The baseline DELCODE dataset included 1079 participants, and only participants with structural MRI data were included in the further selection process (n = 943). This study examined cognitively unimpaired participants; therefore, participants with mild cognitive impairment (MCI) and Alzheimer’s disease (AD) were excluded. In the next steps, only participants with complete data on lifetime musical activity assessment, matching variables, and covariates were included (total: n = 404). Finally, participants with available rs-fMRI and good-quality image data were considered for the matching process (total: n = 394). The final sample after the matching process consisted of n = 130 participants (participants with lifetime musical activity: n = 65, controls: n = 65). FH: family history of Alzheimer’s disease, MRI: magnetic resonance imaging, NC: normal cognition (healthy controls), rs-fMRI: resting-state functional magnetic resonance imaging, SCD: subjective cognitive decline, SES: socioeconomic status.

**S2 Fig. Descriptive characterization of the included participants (n = 65) with lifetime musical activity across the three life stages.** Information is based on participants’ responses about the frequency of musical activity in the Lifetime of Experiences Questionnaire (LEQ). The assessment of lifetime musical activity was - dependent on the participants’ age - assessed across two life stages (13-30 years and 30-65 years) or across three life stages (13-30 years, 30-65 years, 65 years and older).

**S3 Fig. Result of the global (within-network) connectivity analysis.** The ROI-to-ROI connectivity analysis within all three RSN showed no significant group differences. The brain maps (left side) show the DMN (A.), the FPN (C.) and the SAL (E.) networks with all investigated ROIs. Note, ROIs displayed in dark grey specify the network seeds used in our local (seed-to-voxel) connectivity analysis. The corresponding graphs (right side) show the non-significant difference between older participants with lifetime musical activity and matched controls in their global resting-state connectivity (z scores) within the DMN (B.), the FPN (D.) and the SAL (F.). Box plots display the median with 95% confidence intervals, interquartile range with lower (25th) and upper percentiles (75th), and individual data points within each group. Key: ACC: Anterior Cingulate Cortex, AInsula (L): Anterior Insula left, AInsula (R): Anterior Insula right, DMN: Default Mode Network, FC: Functional connectivity, FPN: Fronto-Parietal Network, LP (L): Lateral parietal left, LP (R): Lateral parietal right, LPFC (L): Lateral Prefrontal Cortex left, LPFC (R): Lateral Prefrontal Cortex right, MPFC: Medial Prefrontal Cortex, ROI: Regions-of-interest, PCC: Posterior Cingulate Cortex, PPC (L): Posterior Parietal Cortex left, PPC (R): Posterior Parietal Cortex right, RSN: Resting-State Networks, SAL: Salience Network.

**S1 Table. Network-specific ROIs with their MNI coordinates for the calculation of the global RSFC (for graphical representation see S3 Fig.).** MNI: Montreal Neurological Institute; ROIs: regions-of-interest; RSFC: resting-state functional connectivity.”

## Reviewer comments

### Reviewer 1

#### General comment

**“The current manuscript describes an interesting and relevant cross-sectional study investigating the relationship between lifetime musical activity and resting-state functional connectivity (RSFC) in higherorder cognitive networks in the brain (i.e., the default mode network, frontoparietal, and salience networks).**

**Specifically, Dr. Liebscher and colleagues assessed n=130 cognitive unimpaired older adult individuals (age=>60 years) from the longitudinal DZNE-DELCODE cohort study on risk for dementia and cognitive decline. Individuals, who reported to have participated in musical instrument playing during early, middle, and late life stages (n=65) were compared to matched control subjects, who reported never to have played a musical instrument (n=65), in seed-to-voxel and within-networks RSFC patterns. Individuals with lifetime musical activity displayed higher local RSFC between medial prefrontal cortex, temporal, and frontal regions (right temporal pole and right precentral gyrus) versus control subjects.**

**The authors conclude that playing a musical instrument during life is related to a higher RSFC of medial prefrontal cortex with brain regions involved in higher-order cognitive and motor processes.**

**The manuscript is well-written and relevant. I only have the following minor points:”**

##### Response:

We thank the reviewer for the helpful comments and suggestions and will address those point by point in the next sections. We believe that the changes based on these comments have significantly improved our work.

#### Minor comments

##### Comment #1

**“Abstract**

**Since it is indeed a strength and relevant for interpretation of the observed findings that the two participant groups were matched on several demographic factors, please write these out already in the abstract (i.e., highlight that groups were matched on: age, sex, educational years, crystallized intelligence etc. (cf., Table 1))”**

##### Response:

We thank the reviewer for the suggestion and added this information to the abstract of the revised manuscript:

**Quote from the manuscript, p. 3:** “Participants who reported musical activity during all life stages (*n =* 65) were compared to controls who were matched on demographic and reserve characteristics (including education, intelligence, socioeconomic status, self-reported physical activity, age and sex) and never played a musical instrument (*n =* 65) in local (seed-to-voxel) and global (within-network and between-network) RSFC patterns using pre-specified network seeds”

##### Comment #2

**“Methods, 2.1 Participants:**

**Please provide details on how you specifically operationalized “cognitively unimpaired”? It is unclear how this was assessed with standard neuropsychological tests. Providing information on the criteria for fulfilling this categorization (e.g., performing within certain cut-off values) would be beneficial.”**

##### Response:

We thank the reviewer for this helpful comment. We have revised the manuscript to provide more detailed information on the operationalization of “cognitive unimpairment” or “cognitively unimpaired” older participants and have included the relevant reference to the DELCODE study protocol paper as follows:

**Quote from the manuscript, p. 6:** “Based on our hypothesis, we selected cognitively unimpaired participants from the DZNE-DELCODE baseline cohort [1], including healthy control participants (HC), participants with subjective cognitive decline (SCD), and participants with a family history (FH) of Alzheimer’s disease (AD). To assess cognitive functioning in these participants, the Consortium to Establish a Registry for Alzheimer’s Disease (CERAD) neuropsychological test battery [2] was applied at all assessment sites. Normal or unimpaired cognitive performance was defined by a test performance within –1.5 standard deviations (*SD*) of the age, sex, and education-adjusted norms on all subtests of the CERAD neuropsychological battery. In addition to standard neuropsychological tests, neurological examinations and blood tests were carried out to exclude other diseases and conditions that could affect the participants’ cognition. Further information are provided in the **Supplementary Material** and the DELCODE study protocol [3].”

##### Comment #3

**“Methods, 2.2 Measurements, 2.2.1 Self-reported assessment of musical activity:**

**Although detailed information on the specific categorization process and the one-to one matching procedure are provided in another REF and in the Supplementary Material, the manuscript would benefit from including these descriptions in the main text. Currently, it is unclear how often participants were to have played a musical instrument during life stages to be categorized as ‘having a lifetime history of musical activity’ (i.e., their musical activity ‘frequency’) from the only brief description provided in the current version of chapter 2.2.1. Please include these details from the reference and the supplementary materials here to enhance clarity. Indeed, this would equip the reader with enhanced ability to derive more nuanced conclusions of the identified association.”**

##### Response:

We thank the reviewer for this comment. As noted in the manuscript, the present study is a follow-up study of our previous research work by Böttcher and colleagues [4], where we describe our methodological approach in detail. Following the reviewer’s suggestion, we have revised the present manuscript to provide relevant methodological detail on the categorization process of lifetime musical activity:

**Quote from the manuscript, p 7:** “We used the same categorization scheme as described in detail in our previous study [4]. Briefly, participants answered questions about playing a musical instrument during their lifetime (“How often did you play a musical instrument?”, 6-point Likert-scale: 0: never, 1: less than 1 time per month, 2: 1 time per month, 3: 2 times per month, 4: weekly, 5: daily) across the life stages (13-30 years, 30-65 years, and if applicable ≥ 65 years). Participants who reported that they had played a musical instrument at all life stages, taking into account their respective age, and with a high frequency (≥ 2 times per month) in at least one of these life stages, were categorized as having a lifetime history of musical activity. In contrast, participants who reported to never had played a musical instrument at any given life stage were classified as control group, which was matched in terms of demographic characteristics and reserve proxies (see above). Detailed information on the frequency of playing a musical instrument across the different life stages of each participant in the lifetime musical activity group is provided in our **Supplementary Material** in **Figure S2.**”

##### Comment #4

**“Methods, 2.2.4 Additional measures:**

**How did you specifically operationalize socioeconomic status? Currently, it only says that SES was assessed using the International Socioeconomic Index of Occupational Information. Please specify what the specific items measure.”**

##### Response:

We thank the reviewer for this important comment and have added information about the socioeconomic status (SES) operationalization in the revised manuscript:

**Quote from the manuscript, p 10:** “As described in detail in our previous study [4], the SES was assessed using the International Socioeconomic Index of Occupational Information (ISEI, min. score: 16, max. score: 90) [5], with higher scores corresponding to higher SES. Briefly, the self-reported occupational history of each participant, as assessed by the LEQ across 10 five-year intervals from middle-to-late adulthood (age 30 to 79 years), was coded into occupational categories using the O*Net code system (<https://www.onetonline.org/>) [6]. The O*Net scores were then converted into ISEI scores using fully-automated publicly-available crosswalk procedures that included conversion to Standard Occupational Classification codes (SOC), International Standard Classification of Occupations (ISCO-08) and ISEI calculation (<http://www.harryganzeboom.nl/ISCO08/index.htm>, retrieved: 2021/04). The resulting ISEI scores were averaged across the given time intervals to calculate a mean SES measure for each participant.”

##### Comment #5

**“Discussion:**

**Although the authors have ensured that there were no differences between musical activity participants and matched controls on several demographic variables (cf., Table 1), a more extensive examination of other unaccounted variables in the RSFC association analyses would be beneficial. Please provide a more comprehensive discussion of the potential factors that could also be linked to a history of musical activity and might have influenced the observed differences in local and global functional connectivity patterns (such as potential differences in functional capacity including autonomy, social activity/interpersonal relationships, and occupational functioning that may not be captured by the SES measure applied).”**

##### Response:

We thank the reviewer for this helpful comment. As demonstrated in our manuscript (Table 1), the two groups (participants with lifetime musical activity and matched controls) did not differ significantly on the specific and nonspecific Lifetime of Experiences Questionnaire (LEQ) scores, with the specific LEQ subscore capturing information on educational and occupational attainment between the ages of 13 to > 65. Following the reviewer’s suggestion, we have further assessed possible differences along the suggested characteristics between the two groups with following results:

- **social activities,** assessed using the item on the frequency of social activities (seeing family members; response options: never to daily) of the German version of the Lifetime of Experiences Questionnaire (LEQ-D) [7] averaged across two life stages (13-30 years and 30-65 years) or across three life stages (13-30 years, 30-65 years, 65 years and older), depending on the participants’ age. We found no significant difference between the two groups (*p =* .783).
- **personality traits**, assessed using the German 10-item short form of the Big Five Inventory (BIF-10) [8]. There were no significant differences in the BIF-10 dimensions including openness (*p =* .957), extraversion (*p =* .926), agreeableness (*p =* .856), neuroticism (*p =* .909), conscientiousness (*p =* .606) between the two groups.
- **psychological characteristics**, assessed using the German 15-item short form of the Geriatric Depression Scale (GDS) [9]. There were no significant differences in the total GDS score (*p =* .127) between the two groups.
- **dietary habits**, assessed using body mass index (BMI) of each participant. There were no significant differences in the participants’ BMI (*p =* .305) between the two groups.

The manuscript was revised to include this information as follows:

**Quote from the manuscript, p 12:** “In a post-hoc assessment, we further compared the two groups across additional characteristics and found no significant difference in social activities (*p =* .783) measured using the LEQ [10], personality traits of openness (*p =* .957), extraversion (*p =* .926), agreeableness (*p =* .856), neuroticism (*p =* .909), and conscientiousness (*p =* .606) measured using the 10-item short form of the Big Five Inventory (BFI-10) [8], subclinical depression (*p =* .127) measured using the 15-item short form of the Geriatric Depression Scale (GDS) [9], and the body mass index (BMI, *p =* .305) as an indicator of dietary habits.”

Further, we have included this as a limitation:

**Quote from the manuscript, p 18:** “We considered a number of potentially confounding variables, including reserve proxies, which is a strength of our study. Nevertheless, other variables may be associated with a history of musical activity and might have influenced differences in local and global functional connectivity patterns, including occupational, lifestyle, psychological and health-related characteristics. Albeit, we assessed several of these characteristics (such as self-reported social activity, personality traits, subclinical depression and nutritional health status) and found no significant differences between the two groups, a comprehensive investigation of variables not included in the present RSFC analyses is warranted.”

##### Comment #6

**“Did the authors consider running sensitivity analyses with respect to individuals, who have reported only engaging in solo instrument performance, as compared to participation in musical bands as part of their lifetime musical activities? It would be of interest to explore this aspect and/or discuss the plausible associations potentially emanating from differences in musical instrument engagement on the identified neural benefits associated with participating in musical activities throughout life in older age.”**

##### Response:

We thank the reviewer for giving us the opportunity to elaborate on this point. Unfortunately, we do not have in detail information on the musical instrument engagement in the DELCODE cohort study. We recognize that more specific information on musical activity such as the type of instrument, setting of performance, and age of acquisition would be necessary and worthwhile to investigate. We have revised our manuscript accordingly to acknowledge this information in the limitation section as follows:

**Quote from the manuscript, p 18-19:** “In the present study, musical activity was measured based on the self-reported frequency of playing a musical instrument over the lifetime, hence, the operationalization of this variable is not as ideal as it could be. More detailed information, e.g. on the type of musical instrument, playing solo or multiple instruments, age of acquisition, and/or participation in music groups, should be collected in cohort studies to better understand the association of these differences in musical instrument engagement on the observed neural benefits associated with participation in musical activity in older age.”

##### Comment #7

**“Additionally, it would be interesting to assess and/or discuss whether differences would emerge when accounting for the specific musical instruments played by the individuals (e.g., potential disparities between individuals engaged solely in vocal performance/singing, individuals exclusively playing instrument like the piano or violin, which additionally require intricate fine motor skills involving both the left and right hands). This would be particularly interesting with regards to the identified nodes of precentral gyrus and superior frontal gyrus found to be involved in motor processing and motor control tasks (cf., lines 362-365).”**

##### Response:

We thank the reviewer for this interesting point. As stated in our response to point #6, there was no data on the type of the musical instrument collected in the DELCODE study. We have added this information to the limitation section of in our revised manuscript on page 17, as described above.

##### Comment #8

**“Please also highlight that the samples sizes were modest and the findings need replication before firm conclusions can be drawn (before specifically highlighting the need for future longitudinal studies).”**

##### Response:

We have added this information to the limitation section:

**Quote from the manuscript, p 18:** “Furthermore, the present sample size was modest. Our findings thus need to be replicated in future studies with larger samples to draw firm conclusions before longitudinal studies with rigorous musical training/intervention programs could provide deeper insights into the investigated relationships.”

##### Comment #9

**“Fig. 1**

**In Fig. 1C (FC cluster 1) and 1E (FC cluster 2), please provide value units on the Y-axis (currently, only values are reported).”**

##### Response:

We thank the reviewer for this suggestion and have added the units on the y-axis in Fig. 1 and updated Fig. S3 in the Supplementary Material.

### Reviewer 2

#### All comments

##### Comment #1

**“I have some methodological concerns about this paper. Here are my comments:**

**1. The preprocessing of MRI is too concise. Please describe clearly what has been done. Since subjects were required to be registered to a template, the registration accuracy is very important for this seed-based analysis. Please demonstrate the quality of registration.”**

##### Response:

We thank the reviewer for giving us the opportunity to elaborate on these points. Please find our answers below:

**With regard to the preprocessing of the MRI data:** For the preprocessing of the functional and structural MRI data of the DELCODE database, we used the default preprocessing pipeline for volume-based analyses (direct normalization to MNI-space) implemented in CONN (version 18b). This pipeline has been used in prior studies including Amir and colleagues [11], Busler and colleagues [12]. In addition, we applied the default denoising pipeline implemented in CONN to the functional imaging data. Both, the default preprocessing and denoising pipelines are described in detail on the official CONN manual [13] and the corresponding online documentation (https://web.conn-toolbox.org/home). We perceive the application of these automated pipelines as a strength of our study, as this procedure increases the reproducibility and transparency of our RSFC analyses in accordance with Good Scientific Practice (GSP) guidelines. The manuscript was revised to describe the default preprocessing and denoising pipelines in more detail as follows:

**Quote from the manuscript, p 7-8:** “The MRI images were preprocessed using the default preprocessing pipeline for volume-based analyses (direct normalization to Montreal Neurological Institute (MNI)-space) of the CONN Functional Connectivity Toolbox version 18b [14] in the graphical user interface, based on SPM12 (Wellcome Department of Cognitive Neurology, London, UK; [www.fil.ion.ucl.ac.uk/spm](http://www.fil.ion.ucl.ac.uk/spm)) and implemented in MATLAB [15]. In brief, this preprocessing pipeline includes functional realignment and unwarping, slice-timing correction, outlier identification, and direct segmentation and normalization of the functional and structural images. The pipeline performs a non-linear transformation of the structural and functional MRI data to MNI space using the unified segmentation approach [16] that is based on the separation of the images in gray matter, white matter, and cerebrospinal fluid (CSF). As a result, the functional and structural images are located in the same space without being explicitly co-registered to each other. We performed a visual quality assurance in CONN to assess the quality of co-registration by checking that the anatomical and functional images are aligned with the MNI template. Lastly, the functional images were smoothed with an 8-mm full-width half-maximum Gaussian kernel. Detailed information on the default preprocessing pipeline is available in the CONN manual [13] and the online documentation (<https://web.conn-toolbox.org/fmri-methods/preprocessing-pipeline>).

We further applied the default denoising pipeline implemented in CONN (version 18b), which combines two steps to remove noise, outliers, and motion artifacts that can cause distortions in the functional MRI data. The first step in this denoising pipeline is a linear regression of potential confounding effects in the Blood Oxygenation Level Dependent (BOLD) signal using Ordinary Least Squares. This is followed by the removal of slow-frequency fluctuations with a band pass-filter of [0.008 – 0.09 Hz]. Detailed information on the default denoising pipeline is available in the CONN manual [13] and the online documentation (https://web.conn-toolbox.org/fmri-methods/denoising-pipeline).”

**With regard to the quality of registration:** We implemented several quality assurance (QA) steps in the preprocessing of the MRI data, as recommended in prior publication [17] and implemented in CONN: (1) Before preprocessing, we checked image dimensions and quality of each participant’s MRI scans. (2) After preprocessing and before starting our RSFC analyses, we checked the quality of co-registration (resp. overlap) between the structural images and the MNI template, the overlap between the functional images and the MNI template, the overlap between the structural and functional images. In addition, we checked the segmentation into gray matter, white matter, and CSF, as recommended by Di and colleagues [17] and in online documentations on CONN (https://github.com/andrewjahn/AndysBrainBook/blob/master/docs/FunctionalConnectivity/CONN_ShortCourse/CONN_06_QA_Checks.rst). (3) We further applied the default denoising pipeline (see above) and checked the uniform distribution of the connectivity values as well as the smoothing of the BOLD time series. Examples of this stepwise QA procedure are shown in Fig. 1.

We did not perform a quantitative QA, as the online documentation of CONN and publications such as by Di and colleagues [17] mainly recommend qualitative QA. We hope the reviewer can agree with our QA procedures. The manuscript was revised to includeinformation on the visual QA procedure as follows:

**Quote from the manuscript, p 8:** “We performed a visual quality assurance in CONN to assess the quality of co-registration by checking that the anatomical and functional images are aligned with the MNI template. Lastly, the functional images were smoothed with an 8-mm full-width half-maximum Gaussian kernel.”

***Fig. 1. Examples of visual quality assurance (QA) of the preprocessing after using the default preprocessing pipeline for volume-based analyses (direct normalization to MNI-space) in CONN.***

***A.*** *Visual quality assurance (QA) of the overlap between structural images and the MNI template after using the CONN default preprocessing pipeline.* ***B.*** *Visual QA of the overlap between functional images and the MNI template after using the CONN default preprocessing pipeline.* ***C.*** *Visual QA of the overlap between structural and functional images after using the CONN default preprocessing pipeline.* ***D.*** *Visual QA of segmentation into gray matter after using the CONN default preprocessing pipeline.* ***E.*** *Visual QA of segmentation into white matter after using the CONN default preprocessing pipeline.* ***F.*** *Visual QA of segmentation into CSF after using the CONN preprocessing pipeline. The CONN online documentation (https://web.conn-toolbox.org/fmri-methods/preprocessing-pipeline) provides detailed information on the steps of the used preprocessing pipeline.* ***Key:*** *CSF: Cerebrospinal fluid, MNI: Montreal Neurological Institute.*

##### Comment #2

**“2. The seed-definition is too arbitrary. I think some studies have suggested the seed location of DMN is in around the PCC area. To confirm the accuracy to these seeds and determine if these seeds could be used to form networks, please generate network map based on these selected seeds.”**

##### Response:

We understand the reviewer’s concerns and thank the reviewer for this comment. The seed locations in our RSFC analyses were based on the custom set of network-specific region-of-interests (ROIs) as implemented in CONN. The standard ROIs provided by CONN were previously derived from an independent component analysis of 497 participants as part of the Human Connectome Project (https://www.nitrc.org/projects/conn/) and have been used in several publications by Benson and colleagues [18], Schwarz and colleagues [19], and Krönke and colleagues [20].The manuscript was revised to provide this information:

**Quote from the manuscript, p 8:** “These ROIs were obtained from an independent component analysis on 497 participants of the Human Connectome Project (<https://www.nitrc.org/projects/conn/>) [14]. For future replication studies, the network-specific ROIs used for the RSFC analysis and corresponding MNI centroid coordinates are provided in the **Supplementary Material**.”

For the local RSFC analyses, we deliberately selected three frontal seeds (one for each RSN) of the custom sets of network-specific ROIs. This was done, because previous research has shown that frontal brain regions are involved in musical activity/training [18, 21, 22], cognitive reserve proxies [23, 24] and cognitive functions in the context of aging and Alzheimer’s disease (AD) [for review see 25, 26-28]. The manuscript was revised to provide this information:

**Quote from the manuscript, p 9:** “The selection of frontal seeds for the RSN was based on previous research, showing an involvement of higher-order frontal regions in musical activity/training [4, 21, 22], cognitive reserve processes [23, 24] and cognitive functions associated with aging and AD [for review see 25, 26-28].”

Following the reviewer’s advice, we additionally generated RSN maps using our data and the frontal seeds that we selected for our local RSFC analyses. Therefore, we conducted seed-to-voxel analyses for each RSN in CONN, similar to our local RSFC analyses, but focused on calculating the average local RSFC rather than comparing the two groups. Subsequently, the resulting RSN maps were extracted and overlaid in FSL image viewer (version 6.0.4) [29] with the corresponding functional RSN masks, as created by the Greicius Lab at Stanford University (https://greiciuslab.stanford.edu/resources) using a brain connectivity algorithm [30]. As shown in Fig. 2, the frontal seeds can be applied to form respective network maps, which was, however, beyond the scope of our study.

**Fig. 2. Overlap between the RSN maps generated using the selected frontal seeds for the local RSFC analyses and the RSN masks of the Greicius Lab at Stanford University.**

***A****. The brain map shows an overlay between our self-generated network map of the DMN (in red; left: sagittal plane, right: axial plane) using the MPFC as a seed and seed-to-voxel connectivity analyses performed in CONN and the dorsal DMN map generated by the Greicius Lab (https://greiciuslab.stanford.edu/resources) at Stanford University (in blue).* ***B.*** *The brain map shows an overlay between our self-generated network map of the SAL (in red; left: sagittal plane, right: axial plane) using the ACC as a seed and seed-to-voxel connectivity analyses performed in CONN and the anterior SAL map generated by the Greicius Lab (https://greiciuslab.stanford.edu/resources) at Stanford University (in blue).* ***C.*** *The brain map shows an overlay between our self-generated network map of the FPN (in red; left: sagittal plane, right: axial plane) using the left LPFC as a seed and seed-to-voxel connectivity analyses performed in CONN and the left and right ECN map generated by the Greicius Lab (https://greiciuslab.stanford.edu/resources) at Stanford University (in blue).* ***Key:*** *ACC: Anterior Cingulate Cortex, DMN: Default Mode Network, ECN: Executive Control Network, FPN: Fronto-Parietal Network, LPFC: lateral prefrontal cortex, MPFC: Medial Prefrontal Cortex, SAL: Salience Network.*

##### Comment #3

**“3. Please give clear definition of local FC and global FC.”**

##### Response:

We thank the reviewer for this advice and have updated and rewritten our manuscript respectively. We hope that the revised explication will make it easier for the readers to understand these two terms:

**Quote from the manuscript, p 8-9:** “In the context of our study, we assessed “local RSFC” as the functional coupling between a central node (i.e. seed region) of each RSN and other brain regions. The local RSFC was computed for each RSN by the a-priori selection of one ROI (or network seed) for each RSN that was of particular interest for our study. More precisely, we chose frontal seeds for each RSN, namely, for the DMN: medial prefrontal cortex seed (MPFC; +1, +55, -3), SAL: anterior cingulate cortex seed (ACC; 0, +22, +35), and FPN: left lateral prefrontal cortex seed (LPFC; -43, +33, +28). A visualization of seed locations is provided in S3 Fig. in the **Supplementary Material**. The selection of frontal seeds for the RSN was based on previous research, showing an involvement of higher-order frontal regions in musical activity/training [4, 21, 22], cognitive reserve processes [23, 24] and cognitive functions associated with aging and AD [for review see 25, 26-28]. Local RSFC values were calculated using the seed-to-voxel connectivity analysis performed in CONN, similar to our previous studies [18, 19]. First-level whole-brain seed-based connectivity maps were generated for each RSN and each participant by calculating Fisher’s r-to-z-transformed correlation coefficients between the mean BOLD time course of each custom seed and the BOLD time course of all other voxels in the whole brain. The individual seed-based connectivity maps for each RSN were then subjected to a second-level analysis (see below).”

“We further assessed "global RSFC" defined as the functional coupling across spatially distant nodes within each RSN. To quantify global RSFC within each RSN (i.e., intra-network RSFC), we used the network-specific ROIs provided by CONN (see **Supplementary Material** and **S1 Table**). The ROI-to-ROI connectivity values were computed for each pair of ROIs belonging to the respective network based on the pairwise BOLD signal correlations, which were then converted to *z-scores* using Fisher’s r-to-z transformation. Using the automated script “*conn_withinbetweenROItest*” implemented in CONN, the average functional connectivity for each RSN and each participant was computed. This measure of intra-network RSFC was extracted from CONN and subjected to statistical analysis.”

##### Comment #4

**“4. Statistical analysis is confusing. In the method section, three different threshold are mentioned as significant. For example, "p-values of < .05 were considered statistically significant", "using a voxel- 277 level threshold of *p <* .005 (uncorrected)", "a cluster extend threshold of *p <* .05 corrected for 278 multiple comparisons using False Discovery Rate". Please clarify the statistical significance level and ensure all the reported results could pass FDR.”**

##### Response:

We thank the reviewer for the attentive reading and raising this question. Indeed, we used different thresholds for defining statistical significance in the original version of our manuscript. For the voxel-wise RSFC analyses, we used a joint threshold procedure including the cluster-forming height threshold (*p <* .005) that filters out noise at the individual voxel level and the cluster extend threshold (*p <* 0.05, FDR-corrected for multiple comparison) ensures that significant brain regions or networks are spatially coherent and not just isolated points. This threshold procedure is acceptable to detect smaller effects and has been applied in our previous RSFC studies [18, 19].

The statistical thresholds used in the voxel-wise fMRI analysis can differ from the typical statistical threshold applied in behavioral data analysis, because thresholds used in neuroimaging research account for the spatial continuity and interrelation of brain regions rather than isolated significance values. In response to the reviewer's comment, we lowered the threshold for the behavioral data analyses to a threshold of *p <* .005 to bring it in line with the statistical procedures in the voxel-wise fMRI analyses. This threshold was advocated/endorsed by a large number of researchers including Benjamin and colleagues [31]. The manuscript was revised as follows:

**Quote from the manuscript, p 10:** “The statistical models carried out in CONN are described below. For those analyses carried out in R, *p*-values of < .005 were considered statistically significant in agreement with Benjamin and colleagues [31].”

##### Comment #5

**“5. Why not also compare the inter-network FC between two groups.”**

##### Response:

We thank the reviewer for raising this interesting idea. Following the reviewer’s suggestion, we have calculated the inter-network RSFC in a similar way that we used to calculate the intra-network RSFC. For this, we used the in CONN implemented automated *„conn_withinbetweenROItest“* script for each RSN combination (DMN-SAL, DMN-FPM, SAL-FPN). This automated script not only evaluates the connectivity values within networks, but also the RSFC connectivity between specific networks, known as intra-network connectivity. The inter-network RSFC was calculated by averaging the ROI-to-ROI connectivity values (*z-scores*) over all given ROIs of each RSN combination for each participant and then extracted for further statistical analyses.

We then computed multiple regression analyses in R to assess whether there were differences in the individual inter-network RSFC between the two groups (participants with lifetime musical activity and matched controls). We added the scanner location as a covariate, as in our other analyses. Our analyses showed no significant differences between the two groups. We have revised the manuscript to include these additional analyses in methods, results and discussion sections as follows:

**Quote from the manuscript, p 9:** “In a post-hoc analysis, we additionally evaluated the global RSFC between the RSN (i.e., inter-network RSFC). We used the above-mentioned network-specific ROIs and the automated script “*conn_withinbetweenROItest*” as implemented in CONN to compute the inter-network RSFC for each possible network combination (DMN-SAL, DMN-FPN, SAL-FPN). The inter-network RSFC was calculated by averaging the ROI-to-ROI connectivity values (*z-scores*) over all given ROIs of each RSN combination for each participant. The resulting measure of inter-network RSFC was subsequently extracted from CONN for statistical analysis.”

**Quote from the manuscript, p 14:** “In a post-hoc evaluation, we further compared the inter-network

RSFC between two groups. We found no significant differences between participants with lifetime musical

activity and controls in the inter-network RSFC of DMN-SAL (β = -0.068, p = .473), DMN-FPN (β = 0.017,

p = .854), and SAL-FPN (β = -0.025, p = .786).”

**Quote from the manuscript, p 17:** “Assessing global RSFC within and between the triple networks (DMN, SAL and FPN) between OA with lifetime musical activity and matched controls, we found no evidence for significant differences between these two groups. The variability in global RSFC within and between higher-order brain networks is likely more extensive compared to local RSFC patterns and may be less prone to be associated with leisure-time musical activity. Notably, a previous pilot training/intervention study in older adults found in global RSFC within the FPN after a 4-month dance movement program compared to waiting list control [32], with dance activity being considered an enriching multimodal leisure activity with physical, cognitive, and social engagement [33]. It might thus be reasonable to assume that the cross-sectional nature of our study may not have sufficient power to detect presumably subtle differences in global RSFC patterns. In addition, more extensive information on the musical skills/experiences might be beneficial to detect a potential link between engagement in lifetime musical activity and global functional coupling within and between higher-order brain networks in OA.”

##### Comment #6

**“6. Image quality is not good.”**

##### Response:

We thank the reviewer for this information and have uploaded a revised version of our Fig. 1 with a higher resolution of 300 dpi.

### Reviewer 3

#### General comment

**“The authors investigated the impact of playing musical instruments on the functional connectivity of cognitive neural networks. Utilizing the DELCODE Study database and employing a one-to-one matching procedure based on described characteristics, the authors compared FPN, DMN, SAL differences, revealing that "Higher RSFC has been related to protective lifestyle factors and preserved cognitive abilities in the context of brain pathology, supporting a contribution of these neural correlates to cognitive reserve". As suggested by the authors, the higher RSFC discovered in this study between higher order cognitive and motor processes may represent neuroprotective correlates underlying musical activity in older adults (OA). However, the study design exhibits certain shortcomings. If possible, it is strongly recommended that the authors supplement essential data.”**

##### Response:

We thank the reviewer for the attentive reading of our manuscript and for the expert recommendations and questions, which we will address in the following sections.

#### All comments

##### Comment #1

**“1. The issue of sample representativeness is raised. The text only mentions a one-to-one matching procedure, which introduces a considerable arbitrary selection component. It is strongly advised that the authors employ a 1:1 randomization approach to select samples, thus better eliminating differences in confounding factors (including those mentioned in the text, but not limited to only those) between the two groups.“**

##### Response:

We thank the reviewer for this important comment. As this study is a follow-up study of Böttcher and colleagues [4], we adopted and maintained the one-to-one matching procedure in the present study. We have included the limitations of the one-to-one matching procedure in the limitation section (see below). Following the suggestion of reviewer 1, point #5, we have further assessed possible differences among several characteristics including social activities, personality traits, psychological characteristics, and dietary habits between the two groups with null results.

**Quote from the manuscript, p 18:** “We used a one-to-one matching procedure to assign a matched control group to participants with lifetime musical activity. However, this procedure may introduce a potential arbitrary selection bias, which could affect the overall representativeness of the sample. In future or replication studies, a 1:1 randomization approach should rather be used for sample selection to better account for differences in confounding factors.”

##### Comment #2

**“2. It is strongly recommended that the authors supplement data on cognitive function, executive function, emotional changes, and relevant behavioral differences between the two groups in addition to the differences in resting-state brain functional connectivity. Calculating the correlation between brain functional connectivity differences and behavioral differences and conducting mediation analysis models or pathway analysis is preferable to establish persuasive evidence. It is crucial to demonstrate the specific aspects of cognitive function differences caused by the impact of playing musical instruments on the functional connectivity of cognitive neural networks. Therefore, the manuscript requires substantial data supplementation for a major revision.”**

##### Response:

This is an important point and we thank the reviewer for the interesting suggestions. Actually, we have previously investigated the relationship between brain characteristics (brain volume) and multi-domain cognitive functioning in the musically active participants compared to matched controls using the DELCODE cohort [4]. In this initial study, we found that “…participants with musical activity outperformed controls in global cognition, working memory, executive functions, language, and visuospatial abilities…”. There was no significant association between musical activity and volumes in higher-order brain regions, yet, we showed that the association between brain volumes (including frontal regions) and cognitive abilities was enhanced in the musically active older adults, suggesting a more efficient recruitment of neural resources in this group. Based on these findings and recent studies in the field [21, 22], it was the main aim of our present follow-up study, to provide further indication that functional connectivity in higher-order brain regions could be involved in the neural benefits associated with musical activity. In the present study, however, we did not examine the relationship between brain function and cognitive ability, as we believe this would preferably require an independent data set, a larger sample size with variance in brain pathology and cognitive abilities (see below) and more detailed information on musical activity (see also reviewer 1, point #6).

Inspired by the reviewer’s suggestion, we have conducted following exploratory analysis: As stated in our manuscript (p 5), there is evidence to suggest that preserved connectivity in higher-order RSN (namely FPN and SAL) could be related to preserved cognitive abilities in the context of brain pathology [18, 23], pointing towards an involvement of functional connectivity in reserve and resilience processes [for review see 34]. Following the approach described by Benson and colleagues [18], we have explored a potential compensatory or protective role of the musical activity-related RSFC in the association between vascular brain pathology and cognitive abilities. In our present cohort, we do not find a significant interaction as shown in Fig. 3. However, in contrast to the study by Benson and colleagues [18], our sample included only cognitively unimpaired healthy older adults, so the variance in brain pathology is rather limited.

To sum up, albeit we cannot provide direct evidence in the given cohort of cognitively unimpaired participants, we concur with the view that a history of musical activity might support brain reserve and resilience processes [35], which is an interesting avenue to pursue in future studies. We have down toned the interpretation of our results throughout the manuscript and emphasize the aforementioned hypothesis in several lines in the discussion section as follows:

**Quote from the manuscript, p 15:** “Preserved or enhanced functional connectivity could potentially contribute to better brain health and resilience in OA with a history of musical activity – a hypothesis that should be investigated in future studies.”

**Fig. 3. Results of exploratory moderation analyses assessing a potential compensatory/protective role of musical activity-related RSFC in the association between vascular brain pathology (WMH) and cognitive abilities (global cognition).**

*Musical activity-related RSFC between the DMN seed and distant brain regions did not act as a significant moderator on the association between vascular brain pathology (as measured by WMH, unadjusted for ICV) and cognitive abilities (as measured by global cognition using the multi-domain composite score provided by Wolfsgruber and colleagues [36] from the DELCODE neuropsychological assessment battery).* ***Key:*** *DMN: Default Mode Network, ICV: intracranial volume, RSFC: resting-state functional connectivity, WMH: white matter hyperintensities.*

##### Comment #3

**“3. The study lacks strict control over confounding factors. In addition to the factors mentioned in the text such as Age, Education, Crystallized Intelligence, Sex, Diagnostic group, Lifetime Physical Activity, Current Physical Activity, and LEQ, there may be other potential factors such as underlying diseases, nutritional status, emotional conditions, interpersonal relationships, etc. Adhering to the principle of random sampling is essential, and the sample size should ideally reach several thousand individuals.”**

##### Response:

We thank the reviewer for this important consideration. In response to the reviewer’s requests (see also response to reviewer 1, point #5), we have conducted additional analyses to compare our two groups with respect to other potential confounders including social activities, personality traits, psychological characteristics, and dietary habits. There were no significant differences between the two groups. In addition, we have added information on the DELCODE cohort, indicating that the present sample was carefully selected in terms of a normal neuropsychological, neurological, and general health status. Moreover, neurological examinations and blood tests were carried out in the DELCODE study to exclude relevant diseases and conditions that could affect participants’ cognition. We have revised our manuscript to include the above information as follows:

**Quote from the manuscript, p 6:** “To assess cognitive functioning in these participants, the Consortium to Establish a Registry for Alzheimer’s Disease (CERAD) neuropsychological test battery [2] was applied at all assessment sites. Normal or unimpaired cognitive performance was defined by a test performance within –1.5 standard deviations (*SD*) of the age, sex, and education-adjusted norms on all subtests of the CERAD neuropsychological battery. In addition to standard neuropsychological tests, neurological examinations and blood tests were carried out to exclude other diseases and conditions that could affect the participants’ cognition. Further information are provided in the **Supplementary Material** and the DELCODE study protocol [3].”

**Quote from the manuscript, p 12:** “In a post-hoc assessment, we further compared the two groups across additional characteristics and found no significant difference in social activities (*p =* .783) measured using the LEQ [10], personality traits of openness (*p =* .957), extraversion (*p =* .926), agreeableness (*p =* .856), neuroticism (*p =* .909), and conscientiousness (*p =* .606) measured using the 10-item short form of the Big Five Inventory (BFI-10) [8], subclinical depression (*p =* .127) measured using the 15-item short form of the Geriatric Depression Scale (GDS) [9], and the body mass index (BMI, *p =* .305) as an indicator of dietary habits.”

We further have acknowledged the modest sample size in the limitation section of our manuscript:

**Quote from the manuscript, p 18:** “Furthermore, the present sample size was modest. Our findings thus need to be replicated in future studies with larger samples to draw firm conclusions before longitudinal studies with rigorous musical training/intervention programs could provide deeper insights into the investigated relationships.”

## References

1.	Jessen F, Spottke A, Boecker H, Brosseron F, Buerger K, Catak C, et al. Design and first baseline data of the DZNE multicenter observational study on predementia Alzheimer's disease (DELCODE). Alzheimers Res Ther (2018);10(1):15.

2.	Morris JC, Heyman A, Mohs RC, Hughes JP, Van Belle G, Fillenbaum G, et al. The consortium to establish a registry for Alzheimer’s disease (CERAD). Part I. Clinical and neuropsychological assessment of Alzheimer’s disease. Neurology (1989);39:1159-65.

3.	Jessen FS, Annika; Boecker, Henning; Brosseron, Frederic; Buerger, Katharina; Catak, Cihan; Fliessbach, Klaus; Franke, Christiana; Fuentes, Manuel; Heneka, Michael T.; Janowitz, Daniel; Kilimann, Ingo; Laske, Christoph; Menne, Felix; Nestor, Peter; Peters, Oliver; Priller, Josef; Pross, Verena; Ramirez, Alfredo; Schneider, Anja; Speck, Oliver; Spruth, Eike Jakob; Teipel, Stefan; Vukovich, Ruth; Westerteicher, Christine; Wiltfang, Jens; Wolfsgruber, Steffen; Wagner, Michael; Düzel, Emrah. Design and first baseline data of the DZNE multicenter observational study on predementia Alzheimer's disease (DELCODE). Alzheimer's Research & Therapy (2018);10.

4.	Böttcher A, Zarucha A, Köbe T, Gaubert M, Höppner A, Altenstein S, et al. Musical Activity During Life Is Associated With Multi-Domain Cognitive and Brain Benefits in Older Adults. Front Psychol (2022);13:945709.

5.	Ganzeboom, H. B. G., De Graaf PM, Treiman DJ. A standard international socio-economic index of occupational status. Social Science Research (1992);21(1):1-56.

6.	Peterson N, Mumford M, Borman W, Jeanneret P, Fleishman E, Levin K, et al. Understanding Work Using the Occupational Information Network (O*NET): Implications for Practice and Research. Personnel Psychology (2001);54:451-92.

7.	Roeske S, Wolfsgruber S, Kleineidam L, Zulka L, Buerger K, Ewers M, et al. P3-591: A German version of the lifetime of experiences questionnaire (LEQ) to measure cognitive reserve: Validation results from the DELCODE study. Alzheimer's & Dementia (2018);14(7S_Part_25):P1352-P3.

8.	Rammstedt B, John OP. Measuring personality in one minute or less: A 10-item short version of the Big Five Inventory in English and German. Journal of Research in Personality (2007);41:203-12.

9.	Sheikh JI, Yesavage JA. Geriatric Depression Scale (GDS): recent evidence and development of a shorter version. Clin Gerontol (1986):165-73.

10.	Valenzuela MJ, Sachdev P. Assessment of complex mental activity across the lifespan: development of the Lifetime of Experiences Questionnaire (LEQ). Psychol Med (2007);37(7):1015-25.

11.	Amir J, Nair JKR, Del Carpio R, Ptito A, Chen J-K, Chankowsky J, et al. Atypical resting state functional connectivity in mild traumatic brain injury. Brain and Behavior (2021);11.

12.	Busler JN, Coello E, Liao H, Taylor J, Zhao W, Holsen LM, et al. Perceived Stress, Cortical GABA, and Functional Connectivity Correlates: A Hypothesis-Generating Preliminary Study Frontiers in Psychiatry (2022);13.

13.	Nieto-Castanon A. Handbook of functional connectivity magnetic resonance imaging methods in CONN. Boston: Hilbert Press; 2020.

14.	Whitfield-Gabrieli S, Nieto-Castanon A. Conn: A functional connectivity toolbox for correlated and anticorrelated brain networks. Brain Connectivity (2012);2(3).

15.	The MathWorks Inc. Natick. MATLAB. version 9.4.0.813654 (R2018a) ed. Massachusetts2018.

16.	Ashburner J, Friston KJ. Unified segmentation. Neuroimage (2005);26.

17.	Di X, Biswal BB. A functional MRI pre-processing and quality control protocol based on statistical parametric mapping (SPM) and MATLAB. Front Neuroimaging (2023).

18.	Benson G, Hildebrandt A, Lange C, Schwarz C, Kobe T, Sommer W, et al. Functional connectivity in cognitive control networks mitigates the impact of white matter lesions in the elderly. Alzheimers Res Ther (2018);10(1):109.

19.	Schwarz C, Benson GS, Antonenko D, Horn N, Köbe T, Klimecki O, et al. Negative affective burden is associated with higher resting-state functional connectivity in subjective cognitive decline. Sci Rep (2022);12(1):6212.

20.	Krönke K-M, Wolff M, Shi Y, Kräplin A, Smolka MN, Bühringer G, et al. Functional connectivity in a triple-network saliency model is associated with real-life self-control. Neuropsychologia (2020);149.

21.	Leipold S, Klein C, Jäncke L. Musical Expertise Shapes Functional and Structural Brain Networks Independent of Absolute Pitch Ability. The Journal of Neuroscience (2021);41:2496-511.

22.	Ai M, Loui P, Morris TP, Chaddock-Heyman L, Hillman CH, Mcauley E, et al. Musical Experience Relates to Insula-Based Functional Connectivity in Older Adults. Brain Sci (2022);12.

23.	Franzmeier N, Duering M, Weiner M, Dichgans M, Ewers M. Left frontal cortex connectivity underlies cognitive reserve in prodromal Alzheimer disease. Neurology (2017);88(11):1054-61.

24.	Arenaza-Urquijo EM, Landeau B, La Joie R, Mevel K, Mézenge F, Perrotin A, et al. Relationships between years of education and gray matter volume, metabolism and functional connectivity in healthy elders. Neuroimage (2013);83.

25.	Miller EK, Cohen JD. An integrative theory of prefrontal cortex function. Annu Rev Neurosci (2001);24:167-202.

26.	Botvinick MM, Cohen JD, Carter CS. Conflict monitoring and anterior cingulate cortex: an update. Trends Cogn Sci (2004);8(12):539-46.

27.	Euston DR, Gruber AJ, McNaughton BL. The role of medial prefrontal cortex in memory and decision making. Neuron (2012);76(6).

28.	Jobson DD, Hase Y, Clarkson AN, Kalaria RN. The role of the medial prefrontal cortex in cognition, ageing and dementia. Brain Commun (2021);3(3).

29.	Jenkinson M, Beckmann CF, Behrens TEJ, Woolrich MW, Smith SM. FSL. NeuroImage (2012);62(2).

30.	Shirer WR, Ryali S, Rykhlevskaia E, Menon V, Greicius MD. Decoding subject-driven cognitive states with whole-brain connectivity patterns. Cereb Cortex (2012);22:158-65.

31.	Benjamin DJ, Berger JO, Johannesson M, Nosek BA, Wagenmakers E-J, Berk R, et al. Redefine statistical significance. Nat Hum Behav (2018);2.

32.	Balbim GM, Ajilore OA, Erickson KI, Lamar M, Aguiñaga S, Bustamante EE, et al. The Impact of the BAILAMOS™ Dance Program on Brain Functional Connectivity and Cognition in Older Latino Adults: A Pilot Study. J Cogn Enhanc (2021);5.

33.	Kempermann G. Embodied Prevention. Front Psychol (2022);13.

34.	Stern Y, Arenaza-Urquijo EM, Bartrés-Faz D, Belleville S, Cantilon M, Chetelat G, et al. Whitepaper: Defining and investigating cognitive reserve, brain reserve, and brain maintenance. Alzheimer's & dementia : the journal of the Alzheimer's Association (2020);16(9):1305-11.

35.	Jünemann K, Engels A, Marie D, Worschech F, Scholz DS, Grouiller F, et al. Increased functional connectivity in the right dorsal auditory stream after a full year of piano training in healthy older adults. Sci Rep (2023);13.

36.	Wolfsgruber S, Kleineidam L, Guski J, Polcher A, Frommann I, Roeske S, et al. Minor neuropsychological deficits in patients with subjective cognitive decline. . Neurology (2020);95:e1134–e43.
